# Building primary health care teams for universal health coverage in Africa

**DOI:** 10.4102/phcfm.v14i1.3460

**Published:** 2022-04-25

**Authors:** Shabir Moosa

**Affiliations:** 1 Department of Family Medicine and Primary Care, Faculty of Health Sciences, University of the Witwatersrand, Johannesburg, South Africa; 2 Department of Family Medicine, Johannesburg Health District, Gauteng Department of Health, Johannesburg, South Africa; 3 World Organization of Family Doctors, Brussels, Belgium

The Alma Ata Declaration on Primary Health Care (PHC) in 1978 defined PHC profoundly. Comprehensiveness and continuity of care, as well as coordination of referrals, were key attributes of integrated PHC. Unfortunately, African governments function with bureaucratic health systems, where top-down implementation policies, especially selective PHC vertical programmes driven by international agencies, create rigid, large, impersonalised units of PHC.^1^

The World Health Report on PHC in 2008 put forward the ideas of universal health coverage (UHC); service delivery reforms that reorganise healthcare services around people’s needs, integrating public health actions with primary care and leadership reforms (where governments act as custodians of the whole health system, including private sector and not just of the public service). In reorganising PHC, it urged bringing care closer to people with a primary care team (as a hub of coordination) taking care of a well-defined population.^2^ The operational ramework for Astana set out several operational levers including models of care that prioritise primary care and public health functions, as well as the engagement with private providers in new payment systems.^3^ It is unfortunate that the African Regional Office of the World Health Organization (WHO) does not seem to be rigorous in directing governments in Africa with these ideas.

A conceptual framework put forward by the PHC Performance Initiative (PHCPI) digs deeper into service delivery reform. It sets out important elements: population health management including empanelment; facility organisation and management, including team-based care organisation; access; availability of effective PHC services, including provider competence and motivation; as well as high-quality PHC, including first contact care, continuity, comprehensiveness, coordination and person centredness.^4^

Healthcare workers trained in family medicine deliver person-centred care that addresses patient’s problems with a bio-psycho-social approach. Global family medicine also treasures the idea of community orientation, managing resources and lifelong learning. In South Africa, the role of family doctors (with 18 months of online work-based training) is expected to go beyond being a competent clinician to being a change agent, a capability builder, a critical thinker, a community advocate and a collaborator.^5^ Family doctors are expected to work with PHC teams to care for defined populations. However, the way in which PHC is organised in Africa makes it very difficult for family doctors (and PHC teams) to create the high-quality PHC for patients.

Strategic purchasing in UHC reforms across Africa creates the possibility for this to change. Strategic purchasing is about separating public providers from the Ministry of Health and bringing private providers into the equation. Mixed capitation payment (including some fee-for-service and performance payment) can allow small, decentralised and empowered PHC teams to take care of empanelled populations. Empanelment is a foundational component of PHC. It is about assigning populations to facilities or PHC teams with a responsibility to know their assigned population and to deliver coordinated PHC to them. Empanelment allows PHC to move from reactive care oriented around facility visits by patients, to proactive care that leverages the PHC team’s potential to improve the population health. It allows a relationship to be built between the population and their providers.^6^

The African Regional Office of PHC is committed to building PHC teams for UHC in Africa. We call for the development of UHC proposals with strategic purchasing, including public and private providers in Africa. Whilst the average global panel populations constitute 1500, there are some countries where it is larger, for example, Brazil with 3500 people cared for by a doctor, nurse and a set of community health workers (CHWs). Africa can explore larger panels of 10 000–30 000 people per PHC team. We need to define and standardise PHC facilities and human resource nomenclature across Africa. We need to establish the total PHC human resources in each country per population, and then work out the ratios per 10 000. It then becomes possible to explore a PHC team composition per panel appropriate to the country, enough to produce equitable access and quality. This approach can be adapted for all members of the PHC team, including oral health and rehabilitation professionals.

Such an approach cannot work without a commitment to allocate sufficient resources to PHC and to reduce the out-of-pocket expenditure to patients. Countries should develop single funding pools with progressive taxation strategies and social solidarity. However, relying only on domestic funding will be insufficient for UHC without global solidarity, especially from global ‘aid’ agencies. Global health spending is $7.5 trillion per year. To achieve PHC targets globally requires $200 billion (2.6% of $7.5 trillion).^7^ Therefore, global social solidarity contributions of just 2.6% of health spending from the rest of the world can take the world forward dramatically and bring the wealth of health to bear on the poorest, especially in Africa. Global agencies and country leaders must be challenged on this: what are they doing practically to build PHC teams for UHC in Africa?
